# Subacute Oral Toxicity Evaluation of Red Pine Oil in Sprague–Dawley Rats: Safety Profiling for Potential Therapeutic Use

**DOI:** 10.1155/jt/5376656

**Published:** 2026-08-24

**Authors:** Sriwidodo Sriwidodo, Iyan Sopyan, Gofarana Wilar, Cecep Suhandi, Bayu Febram Prasetyo, Mawar Subangkit

**Affiliations:** ^1^ Department of Pharmaceutics and Pharmaceutical Technology, Faculty of Pharmacy, Universitas Padjadjaran, Sumedang West Java, 45363, Indonesia, unpad.ac.id; ^2^ Department of Pharmacology and Clinical Pharmacy, Faculty of Pharmacy, Universitas Padjadjaran, Sumedang West Java, 45363, Indonesia, unpad.ac.id; ^3^ Sub Division of Veterinary Pharmacy, Faculty of Veterinary Medicine and Biomedical Science, Bogor Agricultural University, Bogor West Java, 16680, Indonesia, ipb.ac.id

**Keywords:** NOAEL, oral toxicity, *Pinus densiflora*, red pine oil, Sprague–Dawley rats, subacute study

## Abstract

Red pine oil (*Pinus densiflora*) is a monoterpene‐rich essential oil with antimicrobial, antioxidant, and anti‐inflammatory activities. While commonly used topically, its systemic safety following oral use remains underexplored, limiting its development as an oral therapeutic or supplement. This research aims to evaluate the subacute oral toxicity of red pine oil in Sprague–Dawley rats over 28 days, followed by a 14‐day recovery phase. Male and female rats were assigned to six groups (*n* = 10/group) and administered red pine oil at 90, 270, or 1000 mg/kg body weight/day by oral gavage. All test preparations were diluted in fish oil; control groups received fish oil alone. Parameters evaluated included clinical signs, body weight, hematology, serum biochemistry, organ weights, and histopathology of major organs. Satellite groups assessed reversibility. No mortality, behavioral changes, or clinical signs of toxicity were observed. Red pine oil significantly increased hemoglobin, hematocrit, erythrocyte count, lymphocytes, and platelets in male rats, with values returning to baseline after recovery. Triglyceride levels were reduced in males at mid and high doses. Minor increases in AST and urea in high‐dose females were not accompanied by histological abnormalities. Organ weights and tissue structures remained within normal limits. All observed effects were noncumulative and reversible. Red pine oil was well‐tolerated at doses up to 1000 mg/kg/day, with no systemic or organ‐specific toxicity. The established NOAEL was 1000 mg/kg BW. These findings support its safety for oral use and provide a scientific basis for further pharmacological studies.

## 1. Introduction

Natural products have long served as an important source of pharmacologically active compounds. Among them, essential oils have garnered significant interest due to their diverse bioactivities and widespread acceptance in traditional and complementary medicine [1]. Red pine oil, a volatile essential oil distilled from the needles and twigs of *Pinus densiflora*, commonly known as Korean red pine, has gained growing attention for its complex chemical composition and therapeutic potential [2]. Rich in monoterpenes such as α‐pinene, β‐pinene, limonene, and bornyl acetate, red pine oil exhibits various biological effects including antimicrobial, antioxidant, anti‐inflammatory, bronchodilatory, and analgesic activities [3–5].

While traditionally used topically or via inhalation, red pine oil is increasingly being investigated for oral administration, particularly in the context of functional food ingredients and herbal supplements [6, 7]. This trend is supported by increasing consumer demand for natural‐origin therapeutics and by the potential for its bioactive components to exert systemic pharmacological effects when absorbed through the gastrointestinal tract [8, 9]. For instance, α‐pinene has demonstrated gastroprotective and anxiolytic properties, while limonene has shown hepatoprotective and antitumor activities in preclinical models [10–12].

Despite these promising properties, the safety profile of red pine oil when administered orally remains insufficiently understood. Essential oils contain lipophilic and highly reactive compounds that may pose toxicological risks, particularly at high or prolonged doses [13, 14]. Concerns over gastrointestinal irritation, hepatic biotransformation leading to potentially harmful metabolites, and limited toxicokinetic data emphasize the need for robust preclinical evaluation [15]. Most existing safety data on pine oils pertain to dermal applications, leaving a substantial gap in the literature regarding repeated‐dose oral exposure [16, 17].

To facilitate oral administration in a preclinical setting, red pine oil was diluted in fish oil, which was chosen as a physiologically compatible vehicle. Fish oil enhances the solubility and gastrointestinal absorption of lipophilic compounds and maintains colloidal stability, even in volatile essential oils, while being well‐tolerated in rodents during chronic studies [18–20]. For instance, fish oil‐based nanoemulsions significantly improved oral bioavailability and anti‐inflammatory outcomes in murine models [21]. Additionally, self‐emulsifying systems using fish oil have demonstrated over sevenfold increased solubility of lipophilic drugs like rosuvastatin [22].

To advance the pharmaceutical development and regulatory approval of orally administered red pine oil products, subacute toxicity studies are essential [23]. The Organisation for Economic Co‐operation and Development (OECD) Test Guideline 407 recommends 28‐day repeated‐dose toxicity testing in rodents to assess target organ effects, determine the no‐observed‐adverse‐effect level (NOAEL), and inform future clinical dose escalation [24]. These studies are particularly valuable for identifying subclinical or cumulative toxicity not captured in acute exposure models [25].

The present study aimed to evaluate the subacute oral toxicity profile of red pine oil in Sprague–Dawley rats over a 28‐day period. Doses were selected to reflect therapeutic use and exceed anticipated human exposure levels. All control and treatment groups received fish oil as the base vehicle to ensure consistency and to isolate the effects of red pine oil itself. Parameters including clinical signs, body weight, hematological and biochemical markers, organ weights, and histopathological findings were assessed to determine systemic toxicity and organ‐specific effects. The results are expected to contribute essential safety data for the future development of red pine oil‐based therapeutic or supplemental products.

## 2. Material and Methods

### 2.1. Animals and Experimental Design

This study employed male and female Sprague–Dawley rats (*Rattus norvegicus*), 8 weeks of age, obtained from iRATco Veterinary Laboratory Services (Bogor, Indonesia). All experimental procedures were conducted in accordance with the OECD Test Guideline 407 and were approved by the Ethics Committee of Universitas Padjadjaran, Bandung, Indonesia (Approval No. 858/UN6.KEP/EC/2025). Prior to treatment, animals were acclimatized for 1 week and confirmed to be in good health.

A total of 60 rats (30 males and 30 females) were randomly assigned to six experimental groups (*n* = 10 per group; 5 males and 5 females each), consisting of a normal control group, three treatment groups receiving red pine oil at increasing doses, and two satellite groups for reversibility assessment following treatment discontinuation (Table 1). The group allocation was performed using a simple randomization method. For individual and group‐wise identification, each animal was uniquely marked with nontoxic, water‐resistant marker stains on the tail (re‐applied weekly), supplemented by numbered identification cards attached to each individual cage.

**TABLE 1:** Grouping of Sprague–Dawley rats for 28‐day subchronic oral toxicity study of red pine oil.

Group	Treatment description	Dose (mg/kg BW)	Abbreviation
G1	Normal control (vehicle)	0	NC
G2	Red pine oil, low dose	90	RPO90
G3	Red pine oil, medium dose	270	RPO270
G4	Red pine oil, high dose	1000	RPO1000
G5	Satellite normal control	0	SNC
G6	Satellite red pine oil, high dose	1000	SRPO1000

*Note:* Animals were divided into normal control, treatment (90, 270, and 1000 mg/kg BW/day), and satellite groups (normal and high dose) with equal distribution of males and females (*n* = 5/sex/group).

### 2.2. Test Substance and Dose Preparation

The test material used in this study was red pine oil, a liquid essential oil extracted from *P. densiflora* needles and twigs. It was diluted in fish oil to a final concentration of 5% (w/v), resulting in a 1:20 dilution ratio. Dose levels were selected based on a combination of human equivalent dose (HED) considerations and OECD Test Guideline 407 recommendations to ensure both translational relevance and appropriate toxicological assessment [24, 26]. The low dose was defined as the estimated therapeutic equivalent dose, derived from the traditional human consumption dose. Based on body surface area normalization (*K*_*m*_ factor: rat = 6; human = 37) using the formula HED = Animal Dose × (*K*_*m*_, animal/*K*_*m*_, human), the low dose of 90 mg/kg body weight in rats corresponds to an HED of approximately 14.6 mg/kg.

The high dose was set at 1000 mg/kg body weight, which represents the maximum limit dose recommended by OECD TG 407 for a subacute toxicity study when no severe toxicity is anticipated based on preliminary data. The medium dose (270 mg/kg body weight) was calculated using a constant geometric factor of 3 from the low dose (90 mg/kg × 3), ensuring evenly spaced logarithmic intervals between the low and high doses to properly evaluate dose‐dependent toxicity and identify potential dose–response relationships [24, 25]. These doses correspond to HED values of approximately 43.8 mg/kg (medium dose) and 162.2 mg/kg (high dose).

In addition, available toxicological data on pine‐derived oils suggest relatively low acute toxicity. Previous studies on *Pinus halepensis* seed oil reported no mortality or significant toxic effects even at high doses in rodent models, indicating a wide safety margin [27]. However, subacute toxicity data specific to *P. densiflora* oil remain limited, thereby warranting further investigation.

Rats received oral administration once daily for 28 consecutive days via gastric gavage. The administration volume ranged from 0.25 to 1.0 mL depending on body weight, and dose concentrations were adjusted weekly based on individual weight gain. A fresh stock solution was prepared weekly using the following concentrations:

• 0 mg/mL (control)
• 36 mg/mL (RPO90)
• 108 mg/mL (RPO270)
• 400 mg/mL (RPO1000 and satellite)

### 2.3. Animal Housing and Husbandry

Animals were housed individually in ventilated cages (IVC) with a floor area of 625.5 cm^2^ and a height of 18.7 cm. The cages were made of autoclavable PVC and maintained in a facility equipped with an air handling system (60 ACH), temperature control (22 ± 3°C), and humidity regulation (55%–68%). A 12‐h light/dark cycle was applied throughout the study. Bedding was composed of dust‐free wood shavings and replaced every 3 days. Standard rodent chow (18% crude protein, 5.7% fat) and filtered water were provided *ad libitum*, with fresh replacements provided regularly to ensure unrestricted access. Clinical observations were conducted systematically once daily to monitor for any signs of toxicity, behavioral changes, or mortality. Special attention was directed toward central nervous system (CNS) and autonomic alterations, which included the recording of signs such as tremors, convulsions, gait abnormalities, changes in locomotor activity, lethargy, salivation, lacrimation, piloerection, and pupillary response. Additionally, detailed weekly physical examinations were performed outside the home cage to assess skin, fur, eyes, mucous membranes, and reactions to handling, with individual body weights recorded weekly.

### 2.4. Euthanasia and Sample Collection

On Day 29, animals in the main groups were euthanized, while satellite groups were observed for an additional 14‐day recovery period before sacrifice on Day 43. Blood collection was performed exclusively at these terminal time points (Day 29 for main groups and Day 43 for satellite groups) following an overnight fast. Euthanasia was performed under deep anesthesia using ketamine (100 mg/kg BW) and xylazine (3 mg/kg BW) via intraperitoneal injection [28]. Once deep anesthesia was confirmed via the loss of pedal reflex, whole blood samples were collected via cardiac puncture. Following blood collection, a bilateral thoracotomy was performed to ensure humane termination.

Whole blood was collected into EDTA tubes for hematological analysis and plain tubes for serum separation. Serum samples were used to assess liver and kidney function, metabolic parameters, and lipid profiles. Major organs were harvested for gross pathological and histopathological examination.

### 2.5. Hematology and Serum Biochemistry

Hematological parameters were analyzed using an automated hematology analyzer and included red blood cell (RBC) count, hemoglobin (HGB), hematocrit (HCT), white blood cell (WBC) count, and platelet count.

Serum biochemical analysis was performed using a fully automated analyzer to determine:

• Liver function markers: alanine aminotransferase (ALT) and aspartate aminotransferase (AST);
• Renal function markers: blood urea nitrogen (BUN) and creatinine; and
• Metabolic indicators: total cholesterol, triglycerides, and fasting blood glucose.

### 2.6. Histopathological Examination

Following organ harvesting, tissues including the liver, kidneys, spleen, lungs, heart, and stomach were weighed and fixed in 10% neutral buffered formalin. Samples were processed, embedded in paraffin, sectioned at 5 μm, and stained with hematoxylin and eosin (H&E). Histological slides were evaluated under light microscopy for morphological alterations including inflammation, degeneration, necrosis, and other signs of toxicity.

### 2.7. Statistical Analysis

All quantitative data, including body weights, hematological parameters, serum biochemistry values, and organ weights, were expressed as mean ± standard deviation (SD). Prior to inferential statistical analysis, the distribution of the data was formally evaluated for normality using the Shapiro–Wilk test, and the homogeneity of variances was assessed using Levene’s test. For the main experimental groups (normal control [NC], RPO90, RPO270, and RPO1000), one‐way analysis of variance (ANOVA) followed by Tukey’s post hoc test was used for normally distributed data with homogeneous variances to determine statistically significant differences between groups [29]. For data that violated the assumptions of normality or homogeneity, the nonparametric Kruskal–Wallis test followed by Dunn’s multiple comparisons post hoc test was applied. For the satellite groups (Satellite control [SNC] vs. Satellite RPO1000 [SRPO1000]), pairwise comparisons to evaluate the reversibility of effects were performed using the unpaired Student’s *t*‐test for parametric data or the Mann–Whitney *U* test for nonparametric data [30]. A *p* value < 0.05 was considered statistically significant. All statistical analyses and graphical representations were conducted using the “meta” package (Version 8.0‐2) and core stats packages in R software (Version 4.4.3) via the RStudio interface (Version 2024.12.1 + 563).

## 3. Results

### 3.1. Clinical Observations and Mortality

No signs of toxicity or abnormal behavior were observed throughout the 28‐day treatment and 14‐day recovery periods in any group. Animals appeared alert, with normal grooming, posture, mucosal condition, and excretory behavior. There were no incidents of morbidity or mortality in any treatment or satellite group. General activity, including locomotion and feeding behavior, remained stable and comparable to controls throughout the study.

### 3.2. Body Weight Progression

**FIGURE 1:**
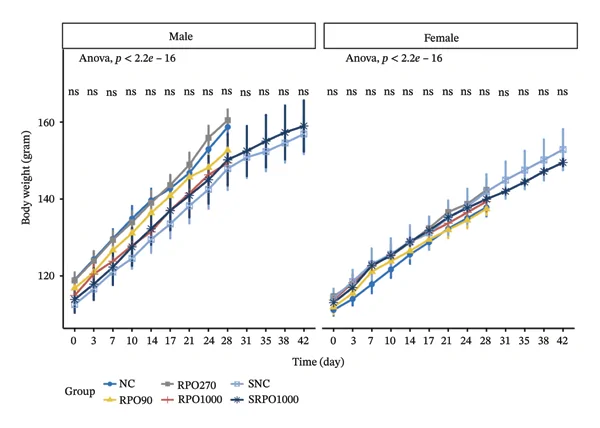
Changes in mean body weight of male and female Sprague–Dawley rats during the 28‐day oral administration of red pine oil and the 14‐day recovery period. Values are presented as mean ± SD. ns = not significant.

All animals exhibited a steady and normal increase in body weight over the 28‐day treatment and 14‐day recovery periods, with no statistically significant differences in mean body weights or growth rates between the control and red pine oil‐treated groups at any weekly interval (Figure 1; detailed numerical data are provided in Table S1 of the Supporting Information). This consistent growth pattern across all doses and both sexes suggests that the oral administration of red pine oil up to 1000 mg/kg body weight did not adversely interfere with the animals’ nutritional status, metabolic efficiency, or general development.

### 3.3. Hematological Parameters

**FIGURE 2:**
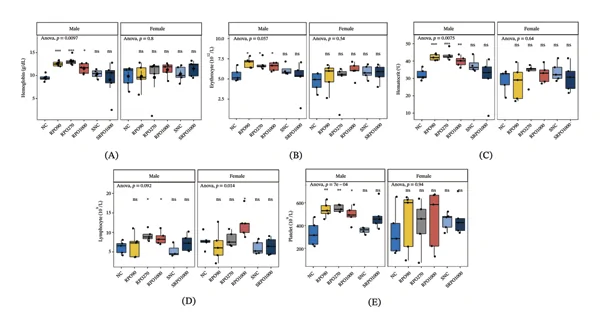
Effects of red pine oil on hematological parameters in male rats: (A) hemoglobin, (B) erythrocyte count, (C) hematocrit, (D) lymphocyte percentage, and (E) platelet count. Values are presented as mean ± SD. *p* < 0.05 (^∗^); *p* < 0.01 (^∗∗^); *p* < 0.001 (^∗∗∗^); ns = not significant.

Red pine oil administration at low, medium, and high doses resulted in statistically significant increases in HGB levels, erythrocyte counts, HCT, lymphocyte percentage, and platelet counts in male rats compared to the control group (*p* < 0.05) (Figure 2, Table S2). These changes were not observed in female rats. Importantly, all values remained within the normal physiological reference ranges and returned to baseline levels in the satellite group after the 14‐day recovery period. Other hematological parameters, including total WBC count, neutrophils, eosinophils, basophils, monocytes, and RBC indices (MCV, MCH, and MCHC) (Figures S1–S6, Tables S3‐S4), were not significantly affected by red pine oil treatment in either sex.

### 3.4. Serum Biochemistry

**FIGURE 3:**
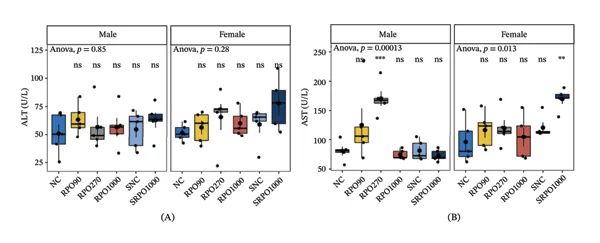
Serum liver enzyme levels following red pine oil administration: (A) alanine aminotransferase (ALT) and (B) aspartate aminotransferase (AST). Values are presented as mean ± SD. *p* < 0.01 (^∗∗^); *p* < 0.001 (^∗∗∗^); ns = not significant.

**FIGURE 4:**
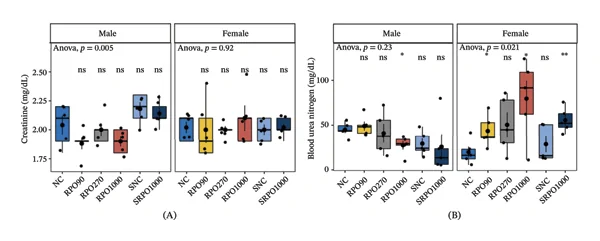
Serum renal function indicators in treated rats: (A) creatinine and (B) blood urea nitrogen (BUN). Values are presented as mean ± SD. *p* < 0.05 (^∗^); *p* < 0.01 (^∗∗^); ns = not significant.

**FIGURE 5:**
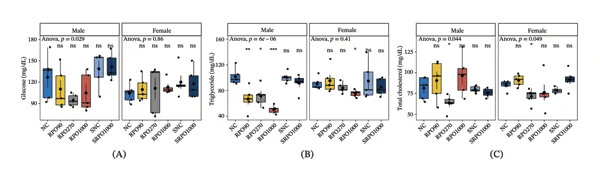
Effects of red pine oil on metabolic parameters: (A) fasting blood glucose, (B) triglycerides, and (C) total cholesterol. Values are presented as mean ± SD. *p* < 0.05 (^∗^); *p* < 0.01 (^∗∗^); *p* < 0.001 (^∗∗∗^); ns = not significant.

Serum biochemical analysis revealed no significant differences in ALT, AST, BUN, or creatinine levels among any of the groups (Figures 3 and 4), indicating that red pine oil did not cause hepatic or renal injury during the study period. However, a statistically significant reduction in triglyceride levels was observed in male rats treated with red pine oil (*p* < 0.01), while total cholesterol and fasting blood glucose levels remained unchanged in both sexes (Figure 5, Table S5). These findings suggest a possible lipid‐lowering effect of red pine oil in male rats, though the effect was not observed in females.

### 3.5. Organ Weights

**FIGURE 6:**
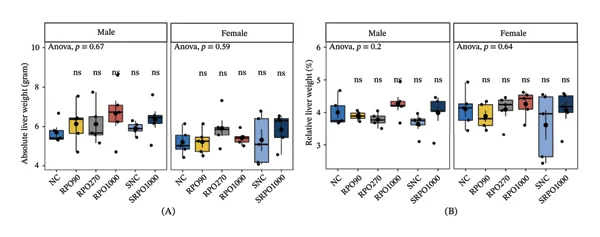
(A) Absolute and (B) relative liver weight following red pine oil treatment. Values are presented as mean ± SD. ns = not significant.

**FIGURE 7:**
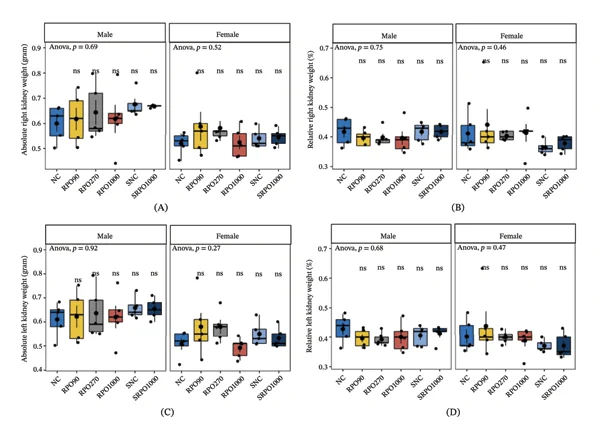
Kidney weight data: (A) Absolute right kidney, (B) relative right kidney, (C) absolute left kidney, and (D) relative left kidney. Values are presented as mean ± SD. ns = not significant.

**FIGURE 8:**
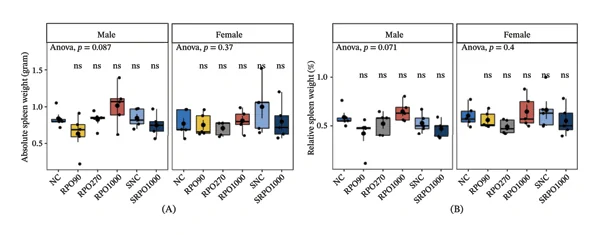
(A) Absolute and (B) relative spleen weight after 28‐day treatment. Values are presented as mean ± SD. ns = not significant.

**FIGURE 9:**
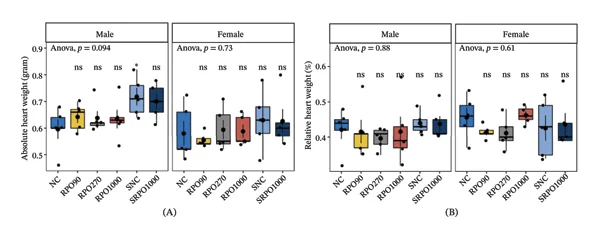
(A) Absolute and (B) relative heart weight in treated and control groups. Values are presented as mean ± SD. *p* < 0.05 (^∗^); ns = not significant.

**FIGURE 10:**
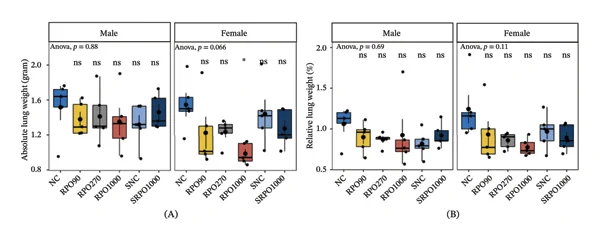
(A) Absolute and (B) relative lung weight following red pine oil administration. Values are presented as mean ± SD. *p* < 0.05 (^∗^); ns = not significant.

There were no statistically significant differences in either absolute or relative organ weights, including liver, kidneys, spleen, heart, and lungs, between control and treated groups in both male and female rats (Figures 6, 7, 8, 9, 10). Organ weights in the satellite groups also remained comparable to the control group, indicating no delayed or persistent effects following red pine oil administration. The comprehensive datasets for both the absolute and relative organ weights are provided in Tables S6 and S7 of the Supporting Information, respectively.

### 3.6. Macroscopic and Histopathological Evaluation

Macroscopic examination of major organs revealed no visible lesions or abnormalities in any group (Figures S7–S11). Organs appeared normal in size, color, and consistency. Histopathological examination confirmed the absence of significant lesions in the liver, kidneys, spleen, lungs, and heart (Figures S12–S16). No necrosis, inflammation, fibrosis, or hemorrhage was detected. Occasional mild degenerative changes were observed in the liver and kidney tissues of some treated animals; however, these findings were not dose‐dependent and were considered incidental and within normal variation. The satellite groups showed normal histological architecture, indicating that any mild tissue alterations were reversible and nonpersistent.

## 4. Discussion

This study presents a comprehensive subacute oral toxicity assessment of red pine oil (*P. densiflora*) in Sprague–Dawley rats over a 28‐day administration period, followed by a 14‐day recovery observation in satellite groups. This is one of the few in vivo studies to evaluate the systemic safety profile of red pine oil when administered orally, especially in relation to hematological, biochemical, and histopathological endpoints. The results confirm that red pine oil, even at the upper dose limit of 1000 mg/kg BW/day, does not elicit mortality, significant behavioral changes, or major organ toxicity, thereby supporting its safe use in therapeutic or nutraceutical contexts.

Importantly, while several toxicological evaluations of pine‐derived oils have been reported, including Korean pine nut oil (*P. koraiensis*), *P. halepensis* seed oil, and pine white oil from *P. pinaster* [27, 31, 32], data specific to *P. densiflora* oil—particularly under subacute exposure conditions—remain limited. Therefore, the present study addresses a critical gap by providing species‐specific safety data that cannot be directly extrapolated from other pine species due to potential differences in phytochemical composition.

A key finding of this study was the statistically significant elevation of hematological parameters, including HGB concentration, HCT, erythrocyte count, lymphocyte percentage, and platelet count, observed predominantly in male rats receiving red pine oil. These changes were not accompanied by adverse clinical signs or pathological evidence of bone marrow stimulation or hemolysis. Rather, they may reflect a physiological upregulation of hematopoiesis, potentially linked to the bioactive components of red pine oil, such as α‐pinene and limonene [33, 34]. These monoterpenes have been reported to exert anti‐inflammatory and antioxidant effects, which may support hematopoietic balance and immune modulation [35]. Similar hematological improvements have been described in rats treated with polyphenolic‐rich plant extracts, including other *Pinus* species. For example, Park et al. demonstrated that *P. densiflora* bark extract enhanced HGB and red cell parameters without hematotoxicity in a 14‐day oral study, highlighting consistency with our findings [36].

In addition to hematologic effects, serum biochemical analysis revealed a notable reduction in triglyceride levels in male rats, particularly at mid and high doses of red pine oil. This hypolipidemic effect may be pharmacologically relevant, considering the role of monoterpenes in modulating hepatic lipid metabolism [37]. Limonene, a major component of red pine oil, has been shown to activate peroxisome proliferator‐activated receptors (PPARs) and enhance fatty acid oxidation, thereby lowering serum triglycerides [38]. Interestingly, this effect was sex‐specific, as it was not observed in female rats, suggesting a potential difference in metabolic or hormonal response between sexes. While cholesterol and glucose levels did not significantly change, the selective reduction in triglycerides aligns with earlier research on *Pinus koraiensis* needle oil, which also demonstrated lipid‐lowering effects in hyperlipidemic animal models [39].

An important consideration in interpreting these biochemical results is the use of fish oil as the vehicle for red pine oil administration. Fish oil is known to possess mild triglyceride‐lowering and anti‐inflammatory properties, which could theoretically influence lipid‐related endpoints [40–42]. However, fish oil was uniformly administered to all groups, including the control, to ensure that any observed changes could be attributed specifically to the red pine oil. The absence of significant lipid or hematological changes in the control group receiving fish oil alone supports the conclusion that the metabolic effects observed were due to red pine oil itself. Furthermore, previous studies have shown that chronic oral administration of fish oil in rodents is well‐tolerated and does not independently induce toxicity, lending further support to the interpretation of these findings [43].

It is important to address the minor elevations in AST and urea observed in high‐dose female rats, which, although statistically significant, were not associated with histopathological evidence of hepatocellular or renal injury. These findings suggest possible transient metabolic stress or adaptive enzyme induction, rather than frank organ toxicity. Histological examination revealed no necrosis, fibrosis, or inflammatory infiltrates in the liver or kidney, supporting the conclusion that these biochemical variations were nonadverse and reversible, especially as they returned to baseline in the satellite groups. These adaptive changes are not uncommon in repeated‐dose studies involving essential oils or terpenoids, many of which induce mild hepatic enzyme shifts without lasting tissue damage [44].

The findings of this study are consistent with prior toxicological evaluations of pine‐derived products. For instance, *Pinus eldarica* aromatic water showed no mortality or organ toxicity up to 22.5 mL/kg in 45‐day oral studies in rats [45]. Similarly, a 13‐week subacute toxicity study of Korean pine nut oil (*P. koraiensis*) in rats reported no systemic toxicity up to 4000 mg/kg BW, with only incidental liver vacuolation, a finding consistent with the incidental hepatic alterations noted in our study [31]. In addition, acute and 28‐day repeated‐dose toxicity studies of cold‐pressed *P. halepensis* seed oil demonstrated no mortality or significant toxic signs in rodents, indicating a wide safety margin [27]. Furthermore, safety assessments conducted by the European Food Safety Authority (EFSA) on pine white oil derived from *Pinus pinaster* also concluded a low toxicity profile under the proposed conditions of use [32]. Nevertheless, it is important to note that differences in species, plant part (e.g., seed oil vs. needle‐derived essential oil), and phytochemical composition—particularly in monoterpene and resin acid content—may significantly influence toxicological outcomes. Therefore, safety data derived from other *Pinus* species cannot be directly extrapolated to *P. densiflora*, underscoring the relevance of the present study.

Importantly, the lack of significant changes in organ weights, food and water consumption, and gross necropsy findings, coupled with normal histological architecture in vital organs, supports the conclusion that red pine oil does not induce systemic or cumulative toxicity under the tested conditions. Even at the highest dose, no irreversible organ damage was evident. The mild histological changes observed in the liver and kidney were random, nondose‐dependent, and fell within the expected background pathology for Sprague–Dawley rats. Such changes are often considered spontaneous or age‐related and are frequently seen even in control groups of long‐term rodent studies.

In a broader context, essential oils have a dual nature: while offering pharmacological benefits, they may pose toxicological concerns due to their high concentration of bioactive compounds [46, 47]. Therefore, robust preclinical safety evaluations such as this are essential. In vitro studies have previously flagged the potential for genotoxicity or cytotoxicity of some essential oils at high concentrations [48, 49]. However, no such adverse outcomes were observed in this in vivo model, underscoring the importance of physiological context when interpreting essential oil safety. Furthermore, *P. densiflora* extracts have shown additional benefits in neuroprotection, anti‐inflammatory activity, and cognitive support in disease models, supporting its growing interest as a multifunctional therapeutic agent [7, 36, 50–52].

Despite these encouraging findings, some limitations of the present study must be acknowledged. The study duration was limited to 28 days, which, while sufficient for identifying early toxicity markers under OECD TG 407, may not detect long‐term or cumulative effects. Although precise quantitative recordings for daily food and water consumption were absent, the lack of treatment‐related changes in growth curves, the absence of muscle‐wasting signs, and the normal physiological ranges of serum protein and metabolic markers collectively indicate that red pine oil did not induce anorexia or nutritional deprivation at the tested doses. Additionally, only standard toxicological endpoints were assessed; future studies should include reproductive toxicity, genotoxicity, and chronic exposure assessments to support a comprehensive safety profile. The observed sex‐based differences in response, both in hematology and lipid metabolism, also warrant further mechanistic exploration, potentially involving hormonal modulation or sex‐specific enzyme expression.

## 5. Conclusion

The 28‐day oral subacute toxicity study of red pine oil in Sprague–Dawley rats established that doses up to 1000 mg/kg BW are safe, with no mortality, clinical pathology, or irreversible organ damage observed. The determined NOAEL of 1000 mg/kg BW aligns with OECD TG 407 criteria and indicates a wide safety margin. Additionally, promising hematopoietic and lipid‐lowering effects, particularly in male rats, point to potential health benefits warranting further exploration. These findings provide robust preclinical evidence supporting the safety and therapeutic promise of red pine oil for oral applications.

## Author Contributions

Conceptualization and methodology: Sriwidodo Sriwidodo, Iyan Sopyan, and Gofarana Wilar; validation: Gofarana Wilar; formal analysis: Bayu Febram Prasetyo and Mawar Subangkit; writing–original draft preparation: Sriwidodo Sriwidodo, Gofarana Wilar, and Cecep Suhandi; writing–review and editing: Iyan Sopyan and Cecep Suhandi; supervision, project administration, and funding acquisition: Sriwidodo Sriwidodo, Cecep Suhandi, and Bayu Febram Prasetyo.

## Funding

This research was funded by the Directorate of Research and Community Service, Directorate General of Research and Development, Ministry of Higher Education, Science and Technology, with financial support through the Doctoral Dissertation Research program, under contract number 093/C3/DT.05.00/PL/2025 and 1669/UN6.3.1/PT.00/2025.

## Disclosure

All authors have read and agreed to the published version of the manuscript.

## Ethics Statement

This study was conducted following ethical approval from the Research Ethics Committee of Universitas Padjadjaran (protocol number: 858/UN6.KEP/EC/2025), granted on October 9, 2025. All animal procedures adhered to national and institutional ethical guidelines for the care and use of laboratory animals. Humane endpoints were predefined, and animals were monitored daily for clinical signs, behavioral changes, and any indications of pain or distress throughout the 28‐day repeated‐dose toxicity study.

Anesthesia and euthanasia were performed when required in accordance with laboratory animal welfare standards. At the end of the study, animals were humanely euthanized for necropsy and histopathological evaluation following OECD Test Guideline 407 procedures. No unexpected severe adverse events requiring early euthanasia occurred during the study.

The study was designed in accordance with the 3R principles: Replacement: Alternative in vitro approaches were evaluated; however, OECD 407 requires in vivo assessment of systemic toxicity following repeated exposure, which could not be adequately addressed by nonanimal methods. Reduction: The minimum number of animals necessary for statistical relevance and compliance with OECD 407 was used. Group sizes were kept as small as possible while maintaining scientific validity. Refinement: Procedures were optimized to reduce discomfort, including adequate acclimatization, gentle handling, environmental enrichment, and routine welfare monitoring. Dose administration was performed using the least stressful method, and any signs of distress were promptly addressed according to predefined humane endpoints.

## Consent

The authors have nothing to report.

## Conflicts of Interest

The authors declare no conflicts of interest.

## Supporting Information

Additional supporting information can be found online in the Supporting Information section.

## Supporting information


**Supporting Information** Supporting Information associated with this article is provided in the file *“Sriwidodo et al_JoT_Supporting Information.docx”*. The document includes Figures S1–S16, presenting extended data and analyses that support the results described in the main text.

## Data Availability

The data that support the findings of this study are available from the corresponding author upon reasonable request.
