# Supplementary material for: Subacute Oral Toxicity Evaluation of Red Pine Oil in Sprague–Dawley Rats: Safety Profiling for Potential Therapeutic Use
Source: J Toxicol. 2026 Aug 24;2026:5376656. doi: 10.1155/jt/5376656 (PMC13504241; doi:10.1155/jt/5376656)
Supplement: Supplementary file 1 [file JT-2026-5376656-s001.docx]

Sriwidodo et al

# Subacute Oral Toxicity Evaluation of Red Pine Oil in Sprague-Dawley Rats: Safety Profiling for Potential Therapeutic Use

Sriwidodo Sriwidodo^1,^*, Iyan Sopyan^1^, Gofarana Wilar^2^, Cecep Suhandi^1^, Bayu Febram Prasetyo^3^, Mawar Subangkit^3^

**SUPPLEMENTARY MATERIALS**

**Table S1.** Body weight progression of male and female Sprague-Dawley rats during the 28-day subacute oral toxicity study and 14-day recovery period.

| **Sex / Group** | **Body Weight (g)** | | | | | | |
| --- | --- | --- | --- | --- | --- | --- | --- |
|  | **Day 0** | **Day 7** | **Day 14** | **Day 21** | **Day 28**  **(Termination)** | **Day 35** | **Day 42 (End of Recovery)** |
| **MALE** | | | | | | | |
| **NC** | 118.97 ± 3.46 | 129.71 ± 5.51 | 139.66 ± 6.74 | 146.78 ± 6.49 | 158.72 ± 5.16 | — | — |
| **RPO90** | 116.80 ± 6.87 | 126.71 ± 6.41 | 136.40 ± 6.72 | 145.68 ± 7.46 | 152.63 ± 9.15 | — | — |
| **RPO270** | 118.83 ± 4.62 | 129.41 ± 5.04 | 139.04 ± 7.00 | 148.94 ± 6.93 | 160.49 ± 6.16 | — | — |
| **RPO1000** | 114.91 ± 6.33 | 123.82 ± 4.93 | 131.50 ± 4.89 | 141.52 ± 4.62 | 149.40 ± 4.77 | — | — |
| **SNC** | 112.55 ± 3.86 | 121.01 ± 5.57 | 129.50 ± 7.76 | 138.23 ± 10.18 | 147.92 ± 12.44 | 152.33 ± 11.41 | 156.90 ± 11.63 |
| **SRPO1000** | 113.87 ± 7.69 | 122.27 ± 10.32 | 132.28 ± 9.34 | 140.92 ± 12.62 | 150.21 ± 14.91 | 155.04 ± 15.19 | 158.98 ± 14.84 |
| **FEMALE** | | | | | | | |
| **NC** | 111.12 ± 3.22 | 117.82 ± 5.20 | 125.57 ± 5.43 | 132.31 ± 4.65 | 137.83 ± 5.16 | — | — |
| **RPO90** | 111.90 ± 4.16 | 121.11 ± 4.68 | 126.52 ± 4.18 | 132.02 ± 4.84 | 137.44 ± 3.53 | — | — |
| **RPO270** | 114.70 ± 4.35 | 123.02 ± 5.16 | 128.68 ± 4.94 | 136.61 ± 5.68 | 142.36 ± 6.28 | — | — |
| **RPO1000** | 113.68 ± 4.63 | 122.42 ± 6.25 | 128.87 ± 5.46 | 133.72 ± 5.47 | 139.35 ± 5.70 | — | — |
| **SNC** | 114.18 ± 5.38 | 123.09 ± 9.06 | 129.03 ± 9.31 | 135.17 ± 9.92 | 141.88 ± 10.18 | 147.54 ± 11.14 | 152.85 ± 11.91 |
| **SRPO1000** | 113.06 ± 4.80 | 122.67 ± 2.63 | 128.87 ± 2.41 | 135.25 ± 1.83 | 139.96 ± 1.41 | 144.41 ± 1.40 | 149.49 ± 2.05 |

**Note:** Data are expressed as mean ± standard deviation (g), with n=5 rats per sex/group.

**Table S2.** Red blood cell parameters, hematocrit, and platelets of male and female Sprague-Dawley rats after 28 days of repeated oral administration of red pine oil and a 14-day recovery period.

| **Sex / Group** | **Hemoglobin (g/dL)** | **Erythrocyte (10^12^/L)** | **Hematocrit (%)** | **Lymphocyte (10^9^/L)** | **PLT (10⁹/L)** |
| --- | --- | --- | --- | --- | --- |
| **MALE** | | | | | |
| NC | 9.50 ± 0.78 | 5.48 ± 0.80 | 32.20 ± 3.21 | 6.15 ± 1.63 | 328.40 ± 108.88 |
| RPO90 | 12.56 ± 0.55 | 7.02 ± 0.62 | 42.22 ± 1.80 | 6.69 ± 3.09 | 539.20 ± 66.06 |
| RPO270 | 13.20 ± 1.06 | 6.80 ± 0.66 | 43.48 ± 3.01 | 9.19 ± 1.36 | 549.40 ± 30.58 |
| RPO1000 | 11.56 ± 1.13 | 6.53 ± 0.51 | 39.90 ± 2.98 | 8.56 ± 1.65 | 493.40 ± 74.00 |
| SNC | 10.35 ± 1.01 | 6.12 ± 0.71 | 37.75 ± 4.34 | 5.34 ± 1.85 | 355.75 ± 29.01 |
| SRPO1000 | 9.08 ± 4.00 | 5.03 ± 2.17 | 29.88 ± 12.67 | 7.32 ± 2.13 | 481.40 ± 118.50 |
| **FEMALE** | | | | | |
| NC | 9.84 ± 2.29 | 4.65 ± 1.15 | 28.92 ± 6.35 | 7.81 ± 2.03 | 338.60 ± 204.06 |
| RPO90 | 9.82 ± 2.69 | 5.26 ± 1.67 | 27.46 ± 9.69 | 6.59 ± 4.06 | 436.60 ± 262.09 |
| RPO270 | 9.58 ± 4.74 | 4.73 ± 2.37 | 33.38 ± 5.60 | 8.25 ± 1.89 | 407.40 ± 219.09 |
| RPO1000 | 11.26 ± 1.55 | 6.06 ± 0.98 | 32.48 ± 4.99 | 12.29 ± 3.56 | 454.00 ± 260.44 |
| SNC | 10.36 ± 1.65 | 5.85 ± 0.82 | 33.72 ± 5.31 | 6.05 ± 1.70 | 438.60 ± 76.81 |
| SRPO1000 | 11.50 ± 1.68 | 5.68 ± 1.09 | 30.44 ± 7.99 | 6.48 ± 2.15 | 456.20 ± 146.34 |

**Note:** Data are expressed as mean ± standard deviation (SD), n = 5 rats per sex/group.

**Table S3.** Total and differential white blood cell counts of male and female Sprague-Dawley rats after 28 days of repeated oral administration of red pine oil and a 14-day recovery period.

| **Sex / Group** | **WBC (10^9^/L)** | **Neutrophil (10^9^/L)** | **Eosinophil (10^9^/L)** | **Basophil (10^9^/L)** | **Monocyte (10^9^/L)** |
| --- | --- | --- | --- | --- | --- |
| **MALE** | | | | | |
| NC | 13.53 ± 5.39 | 4.57 ± 2.13 | 0.04 ± 0.03 | 0.04 ± 0.04 | 0.81 ± 0.70 |
| RPO90 | 12.00 ± 4.72 | 4.88 ± 1.63 | 0.08 ± 0.02 | 0.04 ± 0.04 | 0.43 ± 0.14 |
| RPO270 | 19.65 ± 2.31 | 5.30 ± 1.39 | 0.07 ± 0.03 | 0.04 ± 0.03 | 0.75 ± 0.13 |
| RPO1000 | 18.18 ± 11.06 | 5.68 ± 1.12 | 0.05 ± 0.02 | 0.03 ± 0.04 | 0.54 ± 0.18 |
| SNC | 10.65 ± 1.37 | 3.72 ± 0.57 | 0.06 ± 0.04 | 0.07 ± 0.05 | 0.32 ± 0.17 |
| SRPO1000 | 12.16 ± 2.36 | 5.94 ± 1.19 | 0.05 ± 0.03 | 0.07 ± 0.03 | 0.30 ± 0.33 |
| **FEMALE** | | | | | |
| NC | 12.45 ± 2.62 | 4.17 ± 1.25 | 0.06 ± 0.05 | 0.04 ± 0.03 | 0.42 ± 0.14 |
| RPO90 | 13.99 ± 14.56 | 2.99 ± 1.25 | 0.07 ± 0.02 | 0.05 ± 0.04 | 0.41 ± 0.28 |
| RPO270 | 10.47 ± 5.23 | 4.87 ± 0.83 | 0.06 ± 0.04 | 0.04 ± 0.03 | 0.35 ± 0.18 |
| RPO1000 | 19.70 ± 12.50 | 4.88 ± 1.94 | 0.05 ± 0.04 | 0.06 ± 0.02 | 0.53 ± 0.32 |
| SNC | 12.05 ± 3.69 | 5.93 ± 2.14 | 0.05 ± 0.02 | 0.03 ± 0.02 | 0.67 ± 0.78 |
| SRPO1000 | 11.14 ± 1.38 | 5.40 ± 1.62 | 0.07 ± 0.02 | 0.04 ± 0.02 | 0.35 ± 0.15 |

**Note:** Data are expressed as mean ± standard deviation (SD), n = 5 rats per sex/group.

**Table S4.** Erythrocyte indices (MCV, MCH, and MCHC) of male and female Sprague-Dawley rats after 28 days of repeated oral administration of red pine oil and a 14-day recovery period.

| **Sex / Group** | **MCV (fL)** | **MCH (pg)** | **MCHC (g/dL)** |
| --- | --- | --- | --- |
| **MALE** | | | |
| NC | 59.32 ± 4.75 | 15.46 ± 6.76 | 29.56 ± 1.67 |
| RPO90 | 60.50 ± 5.20 | 17.96 ± 1.41 | 29.70 ± 0.23 |
| RPO270 | 64.10 ± 1.69 | 19.42 ± 0.45 | 30.30 ± 0.60 |
| RPO1000 | 60.10 ± 5.48 | 17.56 ± 2.30 | 29.78 ± 1.04 |
| SNC | 61.70 ± 0.80 | 19.83 ± 0.26 | 27.15 ± 6.00 |
| SRPO1000 | 59.72 ± 2.19 | 18.44 ± 0.72 | 28.88 ± 4.25 |
| **FEMALE** | | | |
| NC | 62.62 ± 4.00 | 21.24 ± 1.01 | 27.14 ± 4.81 |
| RPO90 | 58.54 ± 5.15 | 16.86 ± 7.45 | 33.38 ± 1.65 |
| RPO270 | 62.72 ± 3.35 | 20.48 ± 1.58 | 32.64 ± 2.06 |
| RPO1000 | 57.08 ± 4.40 | 16.24 ± 6.68 | 32.78 ± 1.85 |
| SNC | 59.32 ± 5.24 | 16.02 ± 7.30 | 31.25 ± 1.34 |
| SRPO1000 | 61.06 ± 2.88 | 18.24 ± 0.97 | 29.88 ± 0.72 |

**Note:** Data are expressed as mean ± standard deviation (SD), n = 5 rats per sex/group.

**Table S5.** Serum biochemical profiles of male and female Sprague-Dawley rats after 28 days of repeated oral administration of red pine oil and 14 days of recovery period.

| **Sex / Group** | **Glucose (mg/dL)** | **Total cholesterol (mg/dL)** | **Triglyceride (mg/dL)** | **Creatinine (mg/dL)** | **BUN (mg/dL)** | **ALT (U/L)** | **AST (U/L)** |
| --- | --- | --- | --- | --- | --- | --- | --- |
| **MALE** | | | | | | | |
| **NC** | 127.21 ± 32.08 | 81.40 ± 13.64 | 101.90 ± 12.94 | 2.06 ± 0.19 | 45.06 ± 8.33 | 50.82 ± 18.38 | 80.66 ± 16.72 |
| **RPO90** | 109.97 ± 29.26 | 90.63 ± 23.93 | 66.61 ± 18.73 | 1.87 ± 0.13 | 49.10 ± 11.28 | 63.31 ± 13.82 | 125.10 ± 64.07 |
| **RPO270** | 94.27 ± 8.75 | 63.32 ± 9.78 | 71.49 ± 15.74 | 2.00 ± 0.14 | 41.01 ± 23.22 | 56.63 ± 20.86 | 170.36 ± 28.05 |
| **RPO1000** | 104.72 ± 26.85 | 96.24 ± 24.04 | 50.50 ± 6.32 | 1.89 ± 0.09 | 27.53 ± 10.76 | 56.56 ± 18.18 | 74.51 ± 8.21 |
| **SNC** | 138.73 ± 24.78 | 79.93 ± 4.00 | 100.63 ± 8.52 | 2.16 ± 0.12 | 29.33 ± 13.49 | 54.59 ± 16.72 | 81.18 ± 17.09 |
| **SRPO1000** | 141.49 ± 18.23 | 76.01 ± 5.35 | 91.40 ± 13.92 | 2.14 ± 0.10 | 25.73 ± 31.07 | 62.81 ± 14.91 | 73.03 ± 9.89 |
| **FEMALE** | | | | | | | |
| **NC** | 104.14 ± 13.03 | 84.65 ± 5.96 | 90.68 ± 9.80 | 2.04 ± 0.10 | 19.78 ± 13.72 | 51.51 ± 7.56 | 95.99 ± 37.04 |
| **RPO90** | 109.44 ± 17.17 | 90.85 ± 7.12 | 95.20 ± 20.08 | 2.02 ± 0.25 | 43.60 ± 17.63 | 56.31 ± 13.69 | 116.66 ± 30.70 |
| **RPO270** | 111.35 ± 33.45 | 71.73 ± 9.14 | 83.98 ± 8.05 | 2.02 ± 0.07 | 50.45 ± 31.10 | 65.58 ± 25.65 | 119.99 ± 30.62 |
| **RPO1000** | 112.94 ± 10.18 | 76.24 ± 20.86 | 75.48 ± 5.53 | 2.11 ± 0.26 | 79.66 ± 44.62 | 59.98 ± 12.08 | 104.73 ± 36.39 |
| **SNC** | 119.88 ± 20.55 | 78.52 ± 3.52 | 94.66 ± 29.62 | 2.01 ± 0.12 | 28.76 ± 20.18 | 59.03 ± 16.71 | 120.88 ± 19.22 |
| **SRPO1000** | 117.54 ± 21.51 | 91.59 ± 11.98 | 84.89 ± 13.24 | 2.00 ± 0.08 | 55.73 ± 13.99 | 77.62 ± 22.81 | 169.46 ± 18.42 |

**Note:** Data are expressed as mean ± standard deviation (SD), n = 5 animals per sex/group.

**Table S6.** Absolute organ weights (g) of male and female Sprague-Dawley rats after 28 days of repeated oral administration of red pine oil and 14 days of recovery period.

| **Sex / Group** | **Liver (g)** | **Heart (g)** | **Spleen (g)** | **Right Kidney (g)** | **Left Kidney (g)** | **Lung (g)** |
| --- | --- | --- | --- | --- | --- | --- |
| **MALE** | | | | | | |
| **NC** | 5.70 ± 0.58 | 0.60 ± 0.08 | 0.84 ± 0.12 | 0.60 ± 0.07 | 0.61 ± 0.07 | 1.52 ± 0.33 |
| **RPO90** | 6.14 ± 1.05 | 0.64 ± 0.05 | 0.63 ± 0.26 | 0.62 ± 0.10 | 0.62 ± 0.10 | 1.38 ± 0.19 |
| **RPO270** | 6.12 ± 1.03 | 0.64 ± 0.06 | 0.83 ± 0.11 | 0.64 ± 0.11 | 0.64 ± 0.10 | 1.41 ± 0.31 |
| **RPO1000** | 6.67 ± 1.41 | 0.63 ± 0.08 | 1.02 ± 0.28 | 0.62 ± 0.12 | 0.62 ± 0.10 | 1.35 ± 0.35 |
| **SNC** | 5.90 ± 0.33 | 0.72 ± 0.07 | 0.85 ± 0.12 | 0.68 ± 0.05 | 0.66 ± 0.04 | 1.32 ± 0.25 |
| **SRPO1000** | 6.36 ± 0.92 | 0.70 ± 0.07 | 0.75 ± 0.15 | 0.67 ± 0.00 | 0.65 ± 0.04 | 1.46 ± 0.20 |
| **FEMALE** | | | | | | |
| **NC** | 5.19 ± 0.67 | 0.58 ± 0.10 | 0.77 ± 0.18 | 0.52 ± 0.04 | 0.51 ± 0.05 | 1.55 ± 0.30 |
| **RPO90** | 5.21 ± 0.64 | 0.55 ± 0.03 | 0.75 ± 0.15 | 0.59 ± 0.13 | 0.58 ± 0.13 | 1.22 ± 0.41 |
| **RPO270** | 5.91 ± 0.90 | 0.59 ± 0.08 | 0.71 ± 0.10 | 0.58 ± 0.06 | 0.58 ± 0.06 | 1.23 ± 0.15 |
| **RPO1000** | 5.41 ± 0.35 | 0.59 ± 0.06 | 0.81 ± 0.14 | 0.52 ± 0.06 | 0.49 ± 0.04 | 0.98 ± 0.12 |
| **SNC** | 5.30 ± 1.24 | 0.63 ± 0.11 | 1.00 ± 0.35 | 0.54 ± 0.04 | 0.55 ± 0.05 | 1.44 ± 0.36 |
| **SRPO1000** | 5.84 ± 0.84 | 0.63 ± 0.10 | 0.80 ± 0.25 | 0.54 ± 0.04 | 0.53 ± 0.04 | 1.27 ± 0.22 |

**Note:** Data are expressed as mean ± standard deviation (SD), n = 5 animals per sex/group.

**Table S7.** Relative organ weights (%) of male and female Sprague-Dawley rats after 28 days of repeated oral administration of red pine oil and 14 days of recovery period.

| **Sex / Group** | **Liver (%)** | **Heart (%)** | **Spleen (%)** | **Right Kidney (%)** | **Left Kidney (%)** | **Lung (%)** |
| --- | --- | --- | --- | --- | --- | --- |
| **MALE** | | | | | | |
| **NC** | 4.00 ± 0.43 | 0.42 ± 0.06 | 0.59 ± 0.10 | 0.42 ± 0.05 | 0.43 ± 0.05 | 1.06 ± 0.22 |
| **RPO90** | 3.89 ± 0.13 | 0.42 ± 0.07 | 0.42 ± 0.18 | 0.39 ± 0.03 | 0.40 ± 0.03 | 0.90 ± 0.19 |
| **RPO270** | 3.78 ± 0.21 | 0.40 ± 0.03 | 0.52 ± 0.12 | 0.40 ± 0.03 | 0.39 ± 0.02 | 0.87 ± 0.09 |
| **RPO1000** | 4.27 ± 0.45 | 0.42 ± 0.10 | 0.64 ± 0.10 | 0.40 ± 0.05 | 0.40 ± 0.05 | 0.92 ± 0.45 |
| **SNC** | 3.65 ± 0.33 | 0.44 ± 0.03 | 0.53 ± 0.10 | 0.42 ± 0.03 | 0.41 ± 0.03 | 0.81 ± 0.17 |
| **SRPO1000** | 3.99 ± 0.56 | 0.44 ± 0.05 | 0.47 ± 0.09 | 0.42 ± 0.02 | 0.41 ± 0.03 | 0.92 ± 0.15 |
| **FEMALE** | | | | | | |
| **NC** | 4.11 ± 0.56 | 0.46 ± 0.06 | 0.60 ± 0.11 | 0.41 ± 0.06 | 0.40 ± 0.06 | 1.24 ± 0.39 |
| **RPO90** | 3.89 ± 0.39 | 0.41 ± 0.02 | 0.56 ± 0.08 | 0.44 ± 0.12 | 0.44 ± 0.11 | 0.93 ± 0.37 |
| **RPO270** | 4.09 ± 0.45 | 0.41 ± 0.05 | 0.49 ± 0.07 | 0.40 ± 0.02 | 0.40 ± 0.02 | 0.86 ± 0.10 |
| **RPO1000** | 4.26 ± 0.41 | 0.46 ± 0.03 | 0.64 ± 0.16 | 0.41 ± 0.07 | 0.39 ± 0.05 | 0.77 ± 0.11 |
| **SNC** | 3.61 ± 1.01 | 0.42 ± 0.08 | 0.66 ± 0.20 | 0.36 ± 0.02 | 0.37 ± 0.02 | 0.97 ± 0.22 |
| **SRPO1000** | 4.07 ± 0.59 | 0.44 ± 0.07 | 0.55 ± 0.16 | 0.38 ± 0.03 | 0.37 ± 0.04 | 0.89 ± 0.17 |

**Note:** Data are expressed as mean ± standard deviation (SD) of percentage (%), n = 5 animals per sex/group.

**Figure S1.** Total white blood cell (WBC) count in male rats after 28 days of oral red pine oil administration. Values are presented as mean ± SD. ns = not significant.

**Figure S2.** Percentage of neutrophils in peripheral blood of male rats following red pine oil treatment. Values are presented as mean ± SD. ns = not significant.

**Figure S3.** Percentage of eosinophils in red pine oil-treated male rats. Values are shown as mean ± SD. ns = not significant.

**Figure S4.** Basophil count in peripheral blood following 28-day red pine oil administration. Data are expressed as mean ± SD. ns = not significant.

**Figure S5.** Monocyte percentage in treated and control groups. Values are presented as mean ± SD. ns = not significant.

**Figure S6.** Red blood cell indices in male rats: (A) Mean corpuscular volume (MCV), (B) Mean corpuscular hemoglobin (MCH), and (C) Mean corpuscular hemoglobin concentration (MCHC). Values are presented as mean ± SD. p < 0.05 (*); p < 0.01 (**); ns = not significant.

**Figure S7.** Macroscopic appearance of the liver in control and red pine oil-treated groups. No visible lesions or discoloration were observed.

**Figure S8.** Macroscopic appearance of the kidneys. No signs of enlargement, atrophy, or hemorrhage were noted.

**Figure S9.** Macroscopic view of the spleen. No visible abnormalities were detected across treatment groups.

**Figure S10.** Gross morphology of the heart after 28 days of treatment. Organ shape and color remained normal in all groups.

**Figure S11.** Visual appearance of the lungs. No congestion, consolidation, or discoloration was observed.

**Figure S12.** Histopathological examination of liver tissue (H&E staining, 400×). No evidence of necrosis, inflammation, or fibrosis observed in any group.

**Figure S13.** Histopathological appearance of kidney tissue (H&E staining, 400×). Tubular and glomerular structures remained intact across all groups.

**Figure S14.** Microscopic features of spleen tissue (H&E staining, 400×). White and red pulp structures were normal in all animals.

**Figure S15.** Histological sections of heart tissue (H&E staining, 400×). Myocardial fibers appeared normal with no cellular degeneration.

**Figure S16.** Lung tissue histopathology (H&E staining, 400×). No edema, inflammatory cell infiltration, or alveolar damage observed.
